# The Obstetrical Society of London

**Published:** 1901-10-19

**Authors:** 


### THE OBSTETRICAL SOCIETY OF LONDON.

The last meeting of the Obstetrical Society was occupied in the discussion of a somewhat rare condition, namely, the coincidence of leucæmia and pregnancy. Dr. G. E. Herman read a paper on

ARMY MEDICAL LIBRARY

the subject, reporting a case of his own and dealing with 12 other cases which had been published, in only seven of which, however, was the evidence of the existence of leucæmia regarded as conclusive. The existence of leucæmia in a pregnant woman is evidently a matter to be regarded with much anxiety, and from a consideration of the instances on record Dr. Herman concluded that in such cases the induction of premature labour or abortion was indicated as a therapeutic measure, but only in cases in which the symptoms caused suffering and were aggravated afte the onset of pregnancy.

